# Supplementary material for: mTORC1 and mTORC2 regulate skin morphogenesis and epidermal barrier formation
Source: Nat Commun. 2016 Oct 27;7:13226. doi: 10.1038/ncomms13226 (PMC5095294; doi:10.1038/ncomms13226)
Supplement: Supplementary Information — Supplementary Figures 1-8, Supplementary Tables 1-3 [file ncomms13226-s1.pdf]

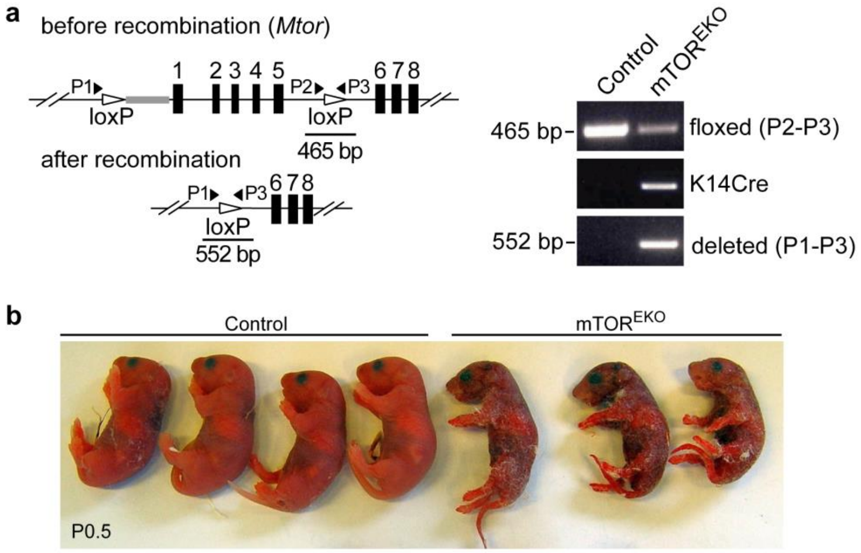

c

Stage	Genotypes of offspring (*Mtor*$^{fl/wt}$*K14Cre* X *Mtor*$^{fl/fl}$)			
	Mtor$^{fl/wt}$	*Mtor*$^{fl/wt}$ *K14Cre*	*Mtor*$^{fl/fl}$	*Mtor*$^{fl/fl}$ *K14Cre*
E14.5-E18.5	20% (n=12)	33% (n=20)	18% (n=11)	28% (n=17)
newborn	28% (n=15)	22% (n=12)	24% (n=13)	26% (n=14)
adult	27% (n=23)	39% (n=33)	34% (n=29)	(n=0)

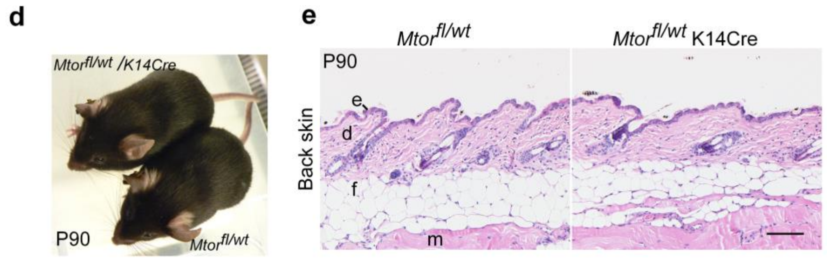

Supplementary Figure 1. Epidermis-specific heterozygous mTOR mutants (*Mtor*$^{EKO/wt}$) are viable and do not show an abnormal skin phenotype.
(a) Left, scheme illustrating the floxed *Mtor* locus and the PCR fragment length before and after recombination. Right, PCR of genomic DNA isolated from embryonic tails showing successful recombination of the floxed region in the *Mtor* locus in the presence of K14-driven Cre. (b) Macroscopic appearance of mTOR^{EKO} newborns and their littermates. (c) Genotypes of offspring from breedings between mTOR$^{fl/wt}$K14Cre and mTOR$^{fl/fl}$. (d) Macroscopic appearance and (e) H&E-stained back skin of mTOR$^{EKO/wt}$ mice. e, epidermis; d, dermis; m, muscle; scale bar represents 50 µm in (e) .

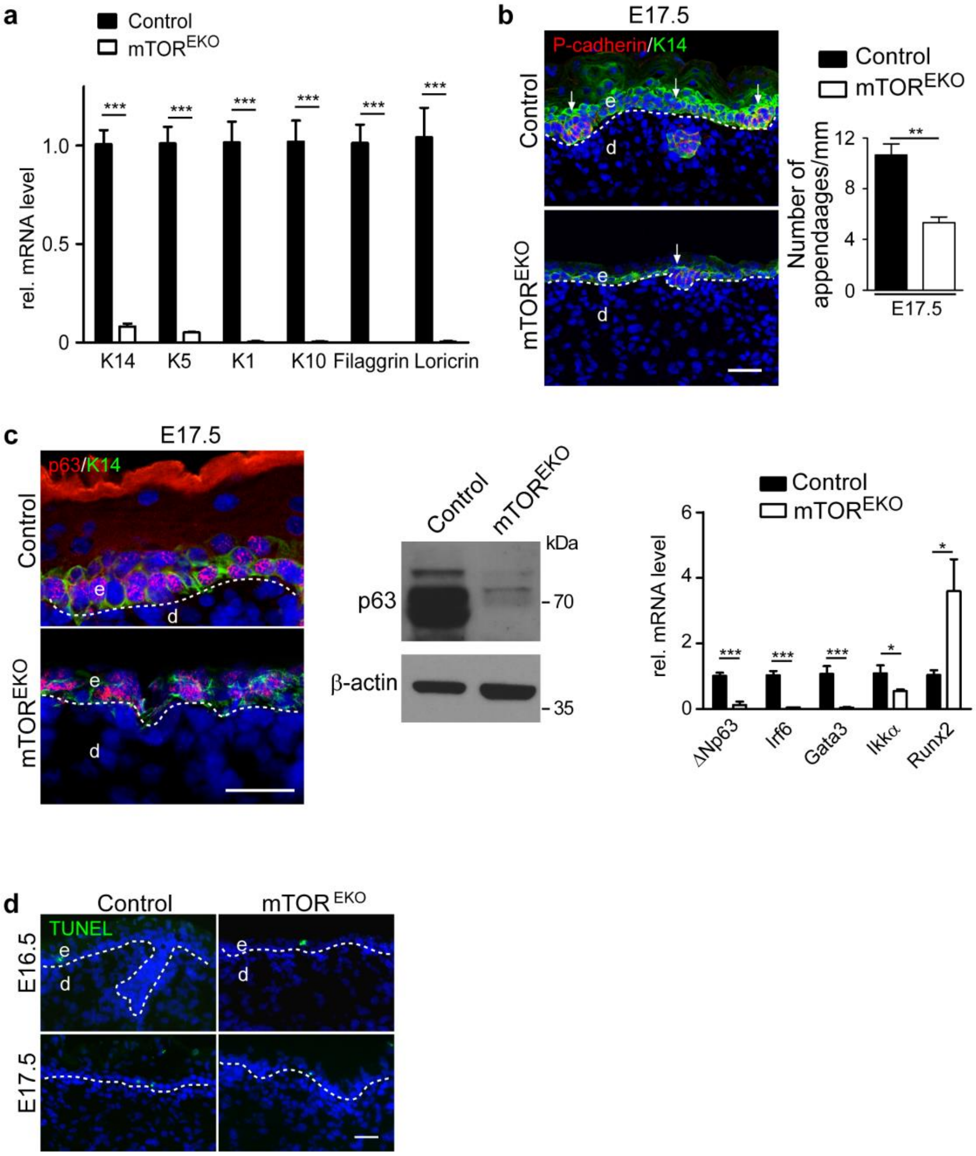

Supplementary Figure 2. mTOR^{EKO} embryos fail to initiate epidermal stratification. (a) qRT-PCR analysis of epidermis in mTOR^{EKO} mice at E17.5 (n = 5 mice/genotype). (b) Representative P-cadherin (red) and K14 (green) double-immunostaining of embryonic back skin (DAPI stain, blue); arrows indicate P-cadherin stained epidermal appendages and quantification of P-cadherin stained skin appendages (*n=5 /genotype*). (c) Left, representative p63 (red) and K14 (green) double-immunostaining of embryonic back skin (DAPI stain, blue). Middle, representative Western blot analysis of p63 expression in E17.5 epidermis. Right, qRT-PCR analysis of ΔNp63 and the target of p63 (Irf6, Gata3, Ikkα and Runx2) expression in mTOR^{EKO} and control epidermis at E17.5 (n = 5 mice/genotype). (d) Representative TUNEL analysis of embryonic back skin. e, epidermis; d, dermis; dashed line indicates basement membrane; scale bar: (b-d) 25 μm; data represents mean±s.d; non-paired t-test was used to calculate P value. *P<0.05, **P<0.01, ***P<0.001.

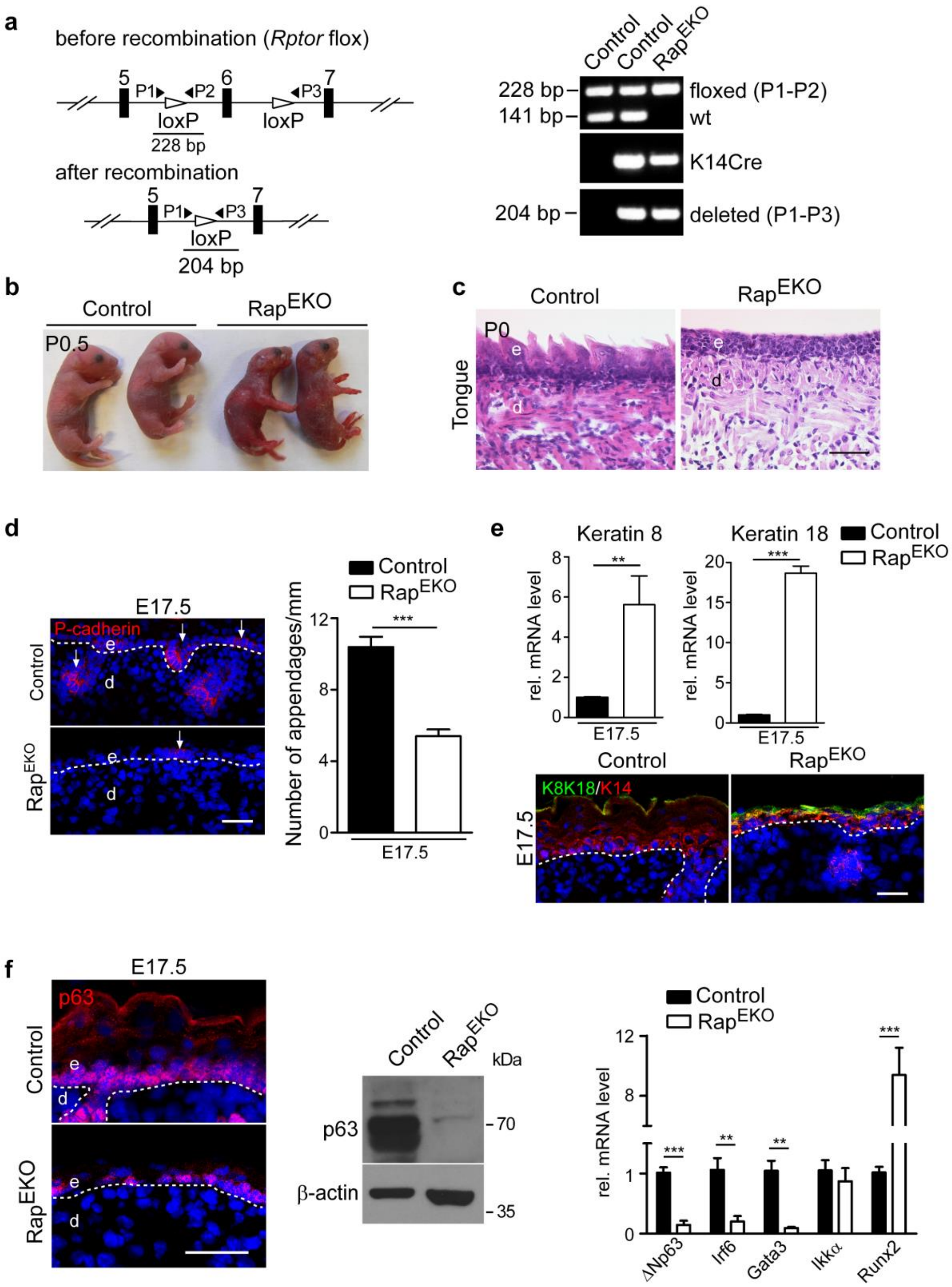

Supplementary Figure 3. Rap^{EKO} mice fail to form a stratified epidermis and oral epithelium during embryonic development.

(a) Left, scheme illustrating the floxed *Rptor* locus and the PCR fragment length before and after recombination. Right, PCR of genomic DNA isolated from embryonic tails showing successful recombination of the floxed region in the *Rptor* locus in the presence of K14-driven Cre. (b) Macroscopic appearance and (c) H&E-stained tongue in Rap^{EKO} newborns. (d) Representative P-cadherin (red) and K14 (green) double-immunostaining of embryonic back skin, arrows indicate p-cadherin stained epidermal appendages and

quantification of P-Cadherin stained skin appendages (*n* = 5 mice/genotype). (e) qRT-PCR analysis and representative immnostaining of K8/18 expression in Rap^{EKO} and control epidermis (*n* = 5/genotype) (f) Left, representative p63 (red) immunostaining of Rap^{EKO} and control back skin (DAPI stain, blue). Middle, representative Western blot analysis of p63 expression in E17.5 epidermis. Right, qRT-PCR analysis of p63 (ΔNp63) and p63 target genes (Irf6, Gata3, Ikkα and Runx2) in Rap^{EKO} and control epidermis at E17.5 (*n* = 5 mice/genotype). e, epidermis; d, dermis; scale bar: (c-f) 25 µm; data represents mean±s.d; non-paired *t*-test was used to calculate *P* value.**P*<0.05, ***P*<0.01, ****P*<0.001.

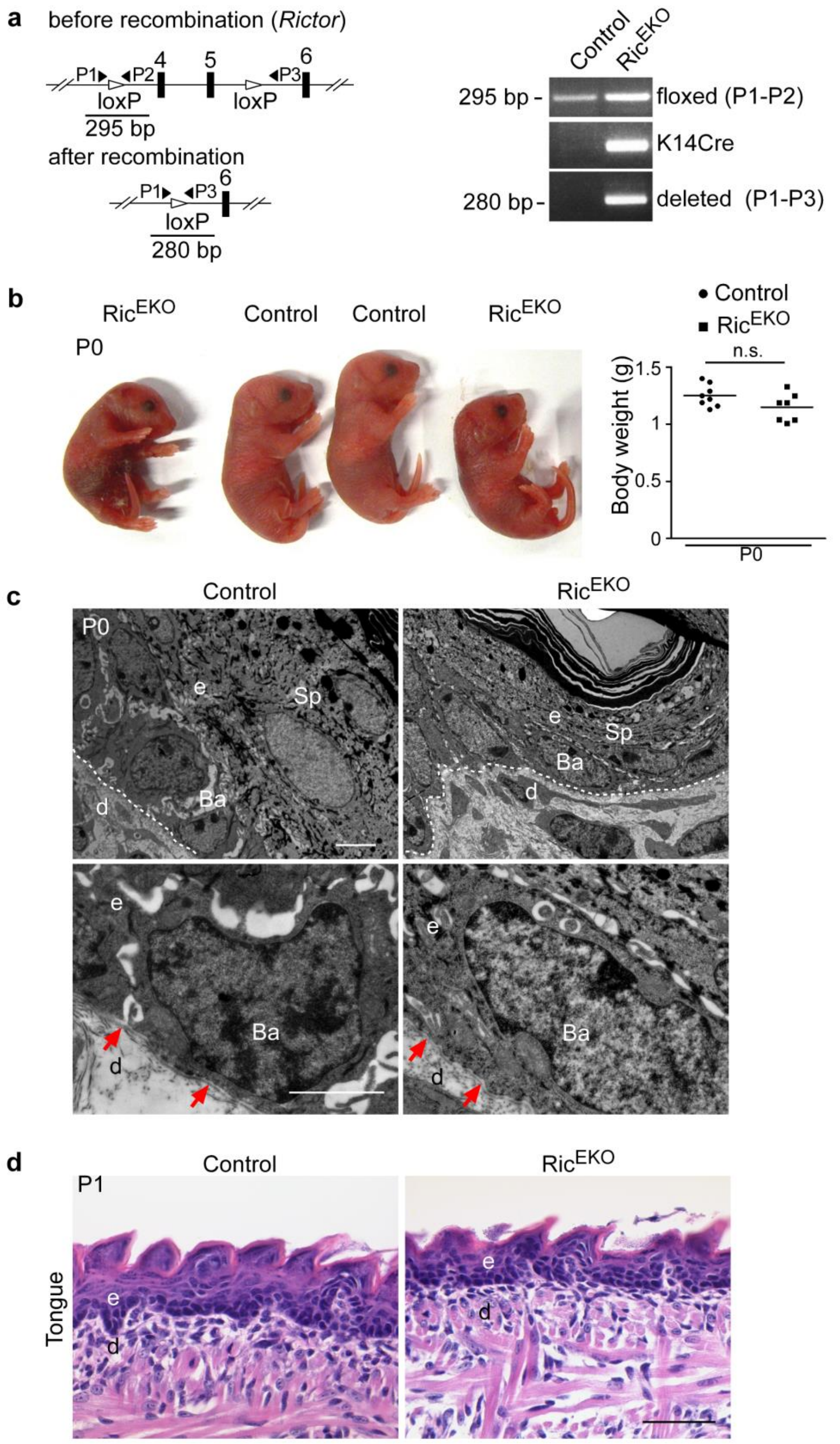

Supplementary Figure 4. Hypoplastic epidermis in Ric^{EKO} mice.
(a) Left, scheme illustrating the floxed *Rictor* locus and the PCR fragment length before and after recombination. Right, PCR of genomic DNA isolated from embryonic tails showing successful recombination of the floxed region in the *rictor* locus in the presence of K14-driven Cre. (b) Left, macroscopic appearance of Ric^{EKO} newborns and their

littermates. Right, body weight in newborns. (c) Representative TEM of newborn skin. The ultrastructure of basal (Ba) and suprabasal (Sp) layers does not reveal obvious alterations in Ric^{EKO} mutants; the basal keratinocytes are well connected to the basal membrane (dotted line and arrows indicates epidermal-dermal border, scale bars: 2 µm. (d) Representative H&E-stained tongue of newborns. e, epidermis; d, dermis; scale bar: 25 µm .

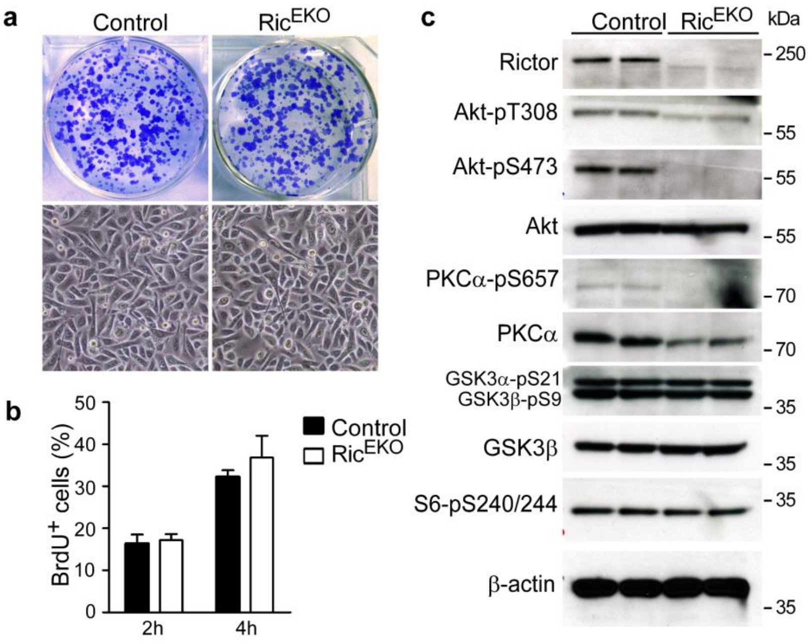

Supplementary Figure 5. mTORC2 signaling does not control autonomous keratinocyte colony forming efficiency or proliferation.
(a) Representative colony-forming assay and cell morphology using primary keratinocytes isolated from controls and Ric^{EKO} mice (the experiment was repeated with 5 mice per genotype). (b) BrdU proliferation assay using primary keratinocytes isolated from controls and Ric^{EKO} mice (*n* = 5 mice/genotype). (c) Representative Western blot analysis of three independent epidermal extract preparations from primary keratinocytes isolated from newborns with antibodies against indicated proteins.

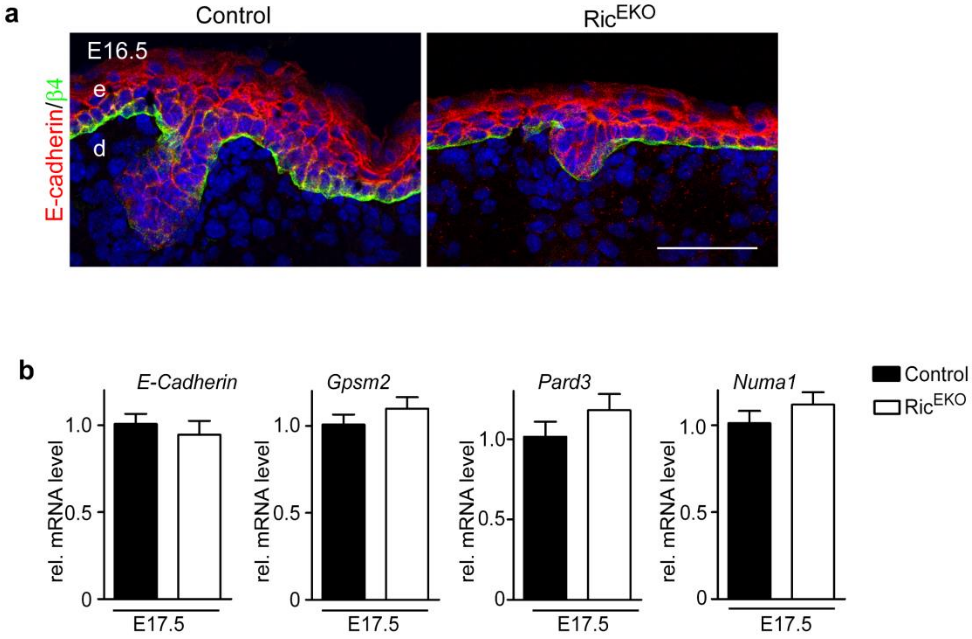

Supplementary Figure 6. Immunostaining of E-cadherin and qRT-PCR analysis of selected genes regulating cell polarity or spindle orientation. (a) Representative E-cadherin (red) and β4-integrin (green) double immunostaining of Ric^{EKO} and control epidermis at E16.5; scale bar: 25 µm; e, epidermis; d, dermis. (b) qRT-PCR analysis of mRNA of E-cadherin, G-protein sigalling modulator 2 (Gpsm2; LGN), partition-defective-3 (Pard3; Par3) and Numa1 (NuMA) in embryonic epidermis (*n* = 5 mice/genotype).

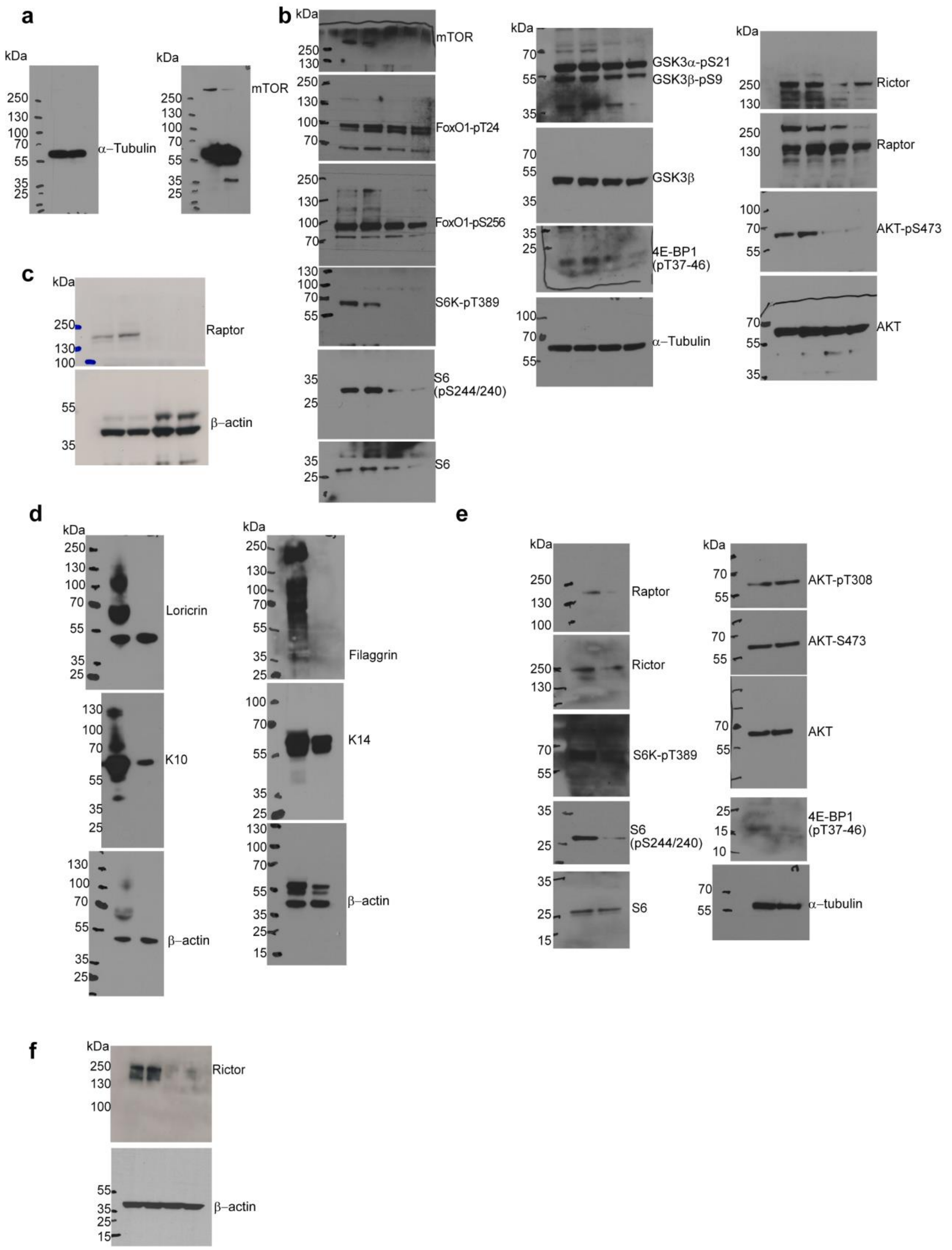

Supplementary Figure 7. Representative not cropped Western blots corresponding to Figure 1a (a), Figure 4b(b), Figure 5a (c), Figure 6f (d), Figure 6g (e) and Figure 7a (f).

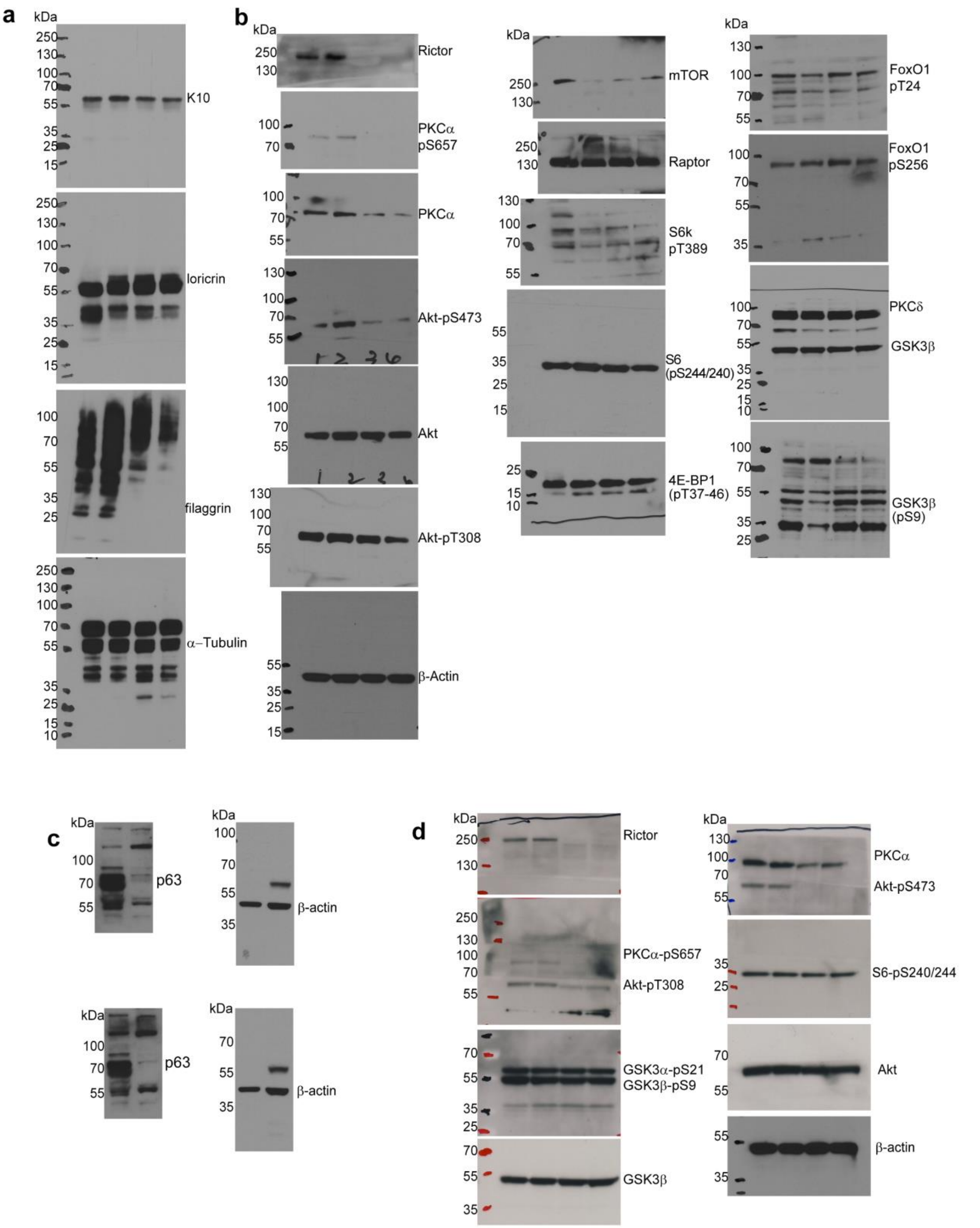

Supplementary Figure 8. Representative not cropped Western blots corresponding to Figure 8d (a), Figure 9a (b), Supplementary Fig 2c and 3f (c), and Supplementary Fig 5c (d).

Supplementary Table 1: Antibodies used for immunostaining

Name	*Catalog number and dilution*	*Source*
mTOR	2983 (1:100)	Cell Signaling, Beverly, MA
S6-pS240/244	5364 (1:2000)	Cell Signaling, Beverly, MA
Akt-pS473	9271 (1:100)	Cell Signaling, Beverly, MA
Loricrin	PRB-145P (1:1000)	Covance, Princeton, New York
Filaggrin	PRB-417P (1:1000)	Covance, Princeton, New York
4E-BP1-pT37/46	2855 (1:100)	Cell Signaling, Beverly, MA
p63	sc-8343 (1:1000)	Santa Cruz Biotechnology
p-cadherin	13-2000Z (1:1000)	Zymed
Keratin14	GP-CK14 (1:50)	Progen, Heidelberg, Germany
Keratin10	PRB-159P (1:1000)	Covance, Princeton, New York
Keratin14	PRB-155P (1:1000)	Covance, Princeton, New York
Keratin15	MS-1068 (1:100)	Thermo Fisher Scientific
K8/18	GP-11 (1:1000)	Progen, Heidelberg, Germany
Cleaved caspase-3	9661 (1:1000)	Cell Signaling, Beverly, MA
Survivin	2808 (1:1000)	Cell Signaling, Beverly, MA
Par3	07-330 (1:1000)	EMD Millipore
LGN	ABT174 (1:2500)	EMD Millipore
β4-integrin	555719 (1:1000)	BD Biosciences
Anti-BrdU	347580 (1:50)	BD Biosciences
E-cadherin	610182 (1:1000)	BD Biosciences
Secondary antibodies		
Anti-rabbit IgG Alexa Fluor® 488	A-11034 (1:500)	Thermo Fisher Scientific
Anti-rabbit IgG Alexa Fluor® 568	A-11011 (1:500)	Thermo Fisher Scientific
Anti- guinea pig Alexa Fluor® 488	A-11073 (1:500)	Thermo Fisher Scientific
Anti-rat IgG Alexa Fluor® 488	A-11006 (1:500)	Thermo Fisher Scientific
Anti-mouse Alexa Fluor® 594	A-11032 (1:500)	Thermo Fisher Scientific
Anti-rabbit IgG HRP	K4003	DAKO

Supplementary Table 2: Primer sequence used for qRT-PCR

Gene	*Forward primer*	*Reverse primer*
GAPDH	CATGTTTGTGATGGGTGTGA	AATGCCAAAGTTGTCATGGA
filaggrin	GGAGGCATGGTGGAACTGA	TGTTTATCTTTTCCCTCACTTCTACATC
loricrin	TCACTCATCTTCCCTGGTGCTT	GTCTTTCCACAACCCACAGGA
E-cadherin	GCTGGACCGAGAGAGTTA	TCGTTCTCCACTCTCACAT
Numa1	GTCAGGCCCCCTTGGAGACT	AGCGGGCCAGAGACTGAGTG
Gpsm2	TCCCCCAACACAGATGAGTTCTT	ATCTTGACCCCTGGCACTTTACA
Pard3	GACAAAGCCGGAAAGGATAAGAAG	CATGCCCCCATACAGCAACTC
keratin14	CGTACTGTTCAGGGTCTGGAG	GCTTCCAGCGATTGTTTCA
keratin10	CGTACTGTTCAGGGTCTGGAG	GCTTCCAGCGATTGTTTCA
keratin5	CAGAGCTGAGGAACATGCAG	CATTCTCAGCCGTGGTACG
keratin8	TATGGGGGACTCACTAGCCC	CAGCTTCCCATCTCGGGTTT
keratin18	AATCGAGGCACTCAAGGAAGAA	GGCATCCACTTCCACAGTCA
ΔNp63	CAAAACCCTGGAAGCAGAAA	GAGGAGCCGTTCTGAATCTG
Irf6	AGGGCTCTGTCATTAATCGAG	TGATTCGGGGCTGCAGTTTC
Gata3	CCGAAACCGGAAGATGTCTA	GTTGAAGGAGCTGCTCTTGG
Ikkα	GTCAGGACCGTGTTCTCAAGG	GCTTCTTTGATGTTACTGAGGGC
Runx2	TCATTCGCCTCACAAAACAAC	CTGCTTGCAGCCTTAAATGA

Supplementary Table 3: Antibodies used for Western blot

Name	*Catalog number and dilution*	*Source*
mTOR	2983 (1:1000)	Cell Signaling, Beverly, MA
S6-pS240/244	5364 (1:2000)	Cell Signaling, Beverly, MA
S6	2317 (1:1000)	Cell Signaling, Beverly, MA
Rictor	2140 (1:1000)	Cell Signaling, Beverly, MA
Raptor	2280 (1:1000)	Cell Signaling, Beverly, MA
Akt-pT308	2965 (1:1000)	Cell Signaling, Beverly, MA
Akt-pS473	9271 (1:1000)	Cell Signaling, Beverly, MA
Loricrin	PRB-145P (1:2000)	Covance, Princeton, New York
Filaggrin	PRB-417P (1:2000)	Covance, Princeton, New York
p70-pT389	9205 (1:1000)	Cell Signaling, Beverly, MA
Akt	9272 (1:1000)	Cell Signaling, Beverly, MA
FoxO1-pT24/32	9463 (1:1000)	Cell Signaling, Beverly, MA
FoxO1-pS256	9461 (1:1000)	Cell Signaling, Beverly, MA
Keratin 14	GP-CK14 (1:1000)	Progen, Heidelberg, Germany
Keratin 10	PRB-159P (1:1000)	Covance, Princeton, New York
GSK3β	9315 (1:1000)	Cell Signaling, Beverly, MA
GSK3α/β-pS9/21	9331 (1:1000)	Cell Signaling, Beverly, MA
PKCα-pS657	sc-12356 (1:1000)	Santa Cruz Biotechnology,
PKCα	2056 (1:1000)	Cell Signaling, Beverly, MA
4E-BP1-pT37/46	9459 (1:1000)	Cell Signaling, Beverly, MA
p63	sc-8343 (1:1000)	Santa Cruz Biotechnology
β-actin (C4)	MAB1501 (1:2000)	Sigma-Aldrich
α-Tubulin (B-5-1-2)	T6074 (1:2000)	Sigma-Aldrich
Secondary antibodies		
Anti-goat-IgG-HRP	P0449 (1:2000)	DAKO
Anti-rabbit-IgG-HRP	P0448 (1:2000)	DAKO
Anti-mouse-IgG-HRP	P0161 (1:2000)	DAKO
Anti-guinea pig -IgG-HRP	P0141 (1:2000)	DAKO
